# Trends in Diverticular Disease Hospitalizations and Racial Disparities in Outcomes Across the United States

**DOI:** 10.7759/cureus.65572

**Published:** 2024-07-28

**Authors:** Fidelis E Uwumiro, Tomilola Olakunde, Adeniyi Fagbenro, Ifeoluwa Fadeyibi, Victory Okpujie, Agatha O Osadolor, Joshua Emina, Grace O Odjighoro, Nonso J Obi, Efe Erhus, Kenechukwu Umenzeakor

**Affiliations:** 1 Internal Medicine, Our Lady of Apostles Hospital, Akwanga, NGA; 2 Internal Medicine, Medical Institute of Sumy State University, Sumy, UKR; 3 Internal Medicine, Bowen University College of Health Sciences, Iwo, NGA; 4 Internal Medicine, Windsor University School of Medicine, Cayon, KNA; 5 Internal Medicine, Central Hospital Benin, Benin, NGA; 6 Family Medicine, University of Benin Teaching Hospital, Benin, NGA; 7 Internal Medicine, University of Benin Teaching Hospital, Benin, NGA; 8 Clinical Sciences, Paelon Memorial Hospital, Lagos, NGA; 9 Internal Medicine, College of Health Sciences, Nnamdi Azikiwe University Teaching Hospital, Nnewi, NGA

**Keywords:** small bowel diverticulosis, racial and ethnic disparities, patient-centered care healthcare disparities, diverticulitis, diverticular diseases

## Abstract

Objective

This study evaluated trends and racial disparities in hospitalization, clinical outcomes, and resource utilization for diverticular disease (DD) between 2017 and 2020.

Methods

We performed a retrospective analysis using the NIS database from 1 January 2017 to 31 December 2020 to study hospitalizations for DD (CCSR code: DIG013). Our primary outcomes were hospitalization rates, all-cause mortality, total charges, and length of stay. Secondary outcomes included in-hospital complications and discharge status. Outcomes were stratified by race and ethnicity (White, Black, Hispanic, Asian or Pacific Islanders and Native Americans). Data were weighted and adjusted for clustering, stratification, and other relevant factors. The normality of the continuous data distribution was confirmed using Kolmogorov-Smirnov, and descriptive statistics were used to summarize variables. Demographic characteristics were compared using χ² and Student’s t-test, with significance set at P<0.05. We used stepwise multivariable logistic regression to estimate adjusted odds ratios for study outcomes by race and ethnicity, controlling for demographic and clinical factors and correcting for multicollinearity. Missing data were treated with multiple imputations, trend analyses were performed using Jonckheere-Terpstra tests, and costs were adjusted for inflation using the GDP price index. Analyses were conducted with Stata 17MP.

Results

A total of 1,266,539 hospitalizations for DD were included for analysis. Approximately 953,220 (75.3%) were White patients and 313,319 (24.7) did not belong to the White race. A total of 747,868 (59%) were women compared to 518,671 (41%) men. Compared to patients who were not of the White race, White patients were younger (63.5 vs. 66.8 years; p<0.001). Hospitalizations for DD increased by 1.2% from 323,764 to 327,770 hospitalizations (2017-2019) and decreased by 11.8% from 327,770 to 289,245 admissions in 2020. Mortality rates were higher among White patients than in those not of the White race (16,205 (1.7%) vs 5,013 (1.6%)). However, no significant difference was observed in mortality odds between both sets of patients (aOR, 0.953; 95% CI 0.881-1.032; P=0.237). Mortality rates showed an uptrend over the study period (4,850 (1.5%) in 2017 to 5,630 (1.9%) in 2020; Ptrend<0.001). DD accounted for 7,016,718 hospital days, 2,102,749 procedures, and US$ 6.8 billion in hospital costs. Mean hospital costs increased from US$54,705 to US$72,564 over the study period (P<0.000). Patients who were not of the White race incurred higher mean hospital charges than White patients ($67,635 ± $6,700 vs $59,103 ± $3,850; P<0.001). Black race correlated with lower odds of bowel perforation, routine home discharge, and bowel resection (P<0.001).

Conclusion

During the study period, hospitalization and mortality rates and resource utilization for DD increased. Patients from other races incurred higher hospital costs than White patients. White Americans were more likely to be discharged to skilled nursing, intermediate care, and other facilities. Additionally, White patients were less likely to develop bowel abscesses compared to patients who were not White. Compared to White American patients, Black patients had fewer DD hospitalizations complicated by bowel perforations or requiring bowel resections. In contrast, DD admissions among Hispanic patients were more likely to require bowel resections than those among White American patients.

## Introduction

The term diverticular disease (DD) includes both asymptomatic diverticulosis and the various complications associated with colonic diverticulosis. Acute diverticulitis, a clinical manifestation of diverticular disease, is a leading gastrointestinal indication for hospitalization in the United States [1]. It imposes a significant burden on individuals and the healthcare system, marking its relevance as a medical condition and a reflective lens into the systemic challenges of healthcare delivery and disparity. Acute diverticulitis is the most common complication (the second being diverticular bleeding) of diverticular disease and a precursor to more severe complications such as perforation, abscess, and fistula formation [2]. Although amenable to conservative management through bowel rest and broad-spectrum antibiotic administration when uncomplicated, complicated diverticulitis may be unresponsive to treatment and result in life-threatening disease [3].

The existing literature shows that the prevalence of diverticular disease may be steadily increasing. In 1998, the United States recorded 3,414 mortalities due to diverticular disease [4]. This resulted in a mortality rate of 2.5 per 100,000 people, placing diverticular disease as the sixth leading cause of death related to gastrointestinal disorders. In 2004, it was responsible for 3,372 deaths out of a total of 2,397,615, representing 0.14% of overall deaths [5]. The current data indicate that annually, in the United States, there are more than 2.7 million outpatient visits and 200,000 inpatient admissions for diverticulitis at a cost of more than $2 billion [6,7].

Although sparse, available knowledge on racial disparities in diverticulosis also reveals that racial disparities are prevalent in the incidence of the disease in the United States. White American individuals typically have higher odds of developing diverticulosis overall. In contrast, Black individuals and Asian/Pacific Islanders tend to have a lower likelihood of developing the disease [8]. However, they have a higher risk of experiencing proximal diverticulosis, a specific form of the disease located at the proximal colon. Among patients with diverticulosis who undergo surgery, the Black race has been independently associated with higher 30-day morbidity and the non-Hispanic/Pacific Islander race has been associated with higher mortality [9].

Using hospitalization data from the Nationwide Inpatient Sample (NIS) database, this study aimed to evaluate temporal trends in the prevalence and outcomes of diverticulitis, juxtaposing them against the racial identities of hospitalized individuals across the United States in 2017-2020.

## Materials and methods

We performed a retrospective analysis using the NIS database from January 1, 2017, to December 31, 2020. The NIS is a publicly accessible database of all payers, approximating a 20% stratified sample of discharges from US community hospitals participating in the Healthcare Cost and Utilization Project. The data include information on each hospitalization, including primary and secondary diagnosis, patient demographics, primary payer, information regarding comorbidities, length of stay, and mortality [10-12]. We queried the NIS database to identify all hospitalizations during the specified period with a primary or secondary diagnosis of diverticular disease using the Clinical Classification Software Refined (CCSR) code DIG013 (diverticulosis and diverticulitis).

We included adult patients aged 18 and older with a primary or secondary diagnosis of diverticular disease who were hospitalized between 2017 and 2020. Inclusion required documented race and ethnicity information to analyze racial disparities. We excluded patients younger than 18, those with incomplete demographic or clinical data, and those hospitalized for disorders other than diverticular disease.

Our primary outcomes of interest included rates and trends in hospitalization, all-cause in-hospital mortality, total hospital charges, and length of hospital stay. Mortality, cost, and hospital length of stay variables were already defined in the NIS. Secondary outcomes included in-hospital complications (including abscess formation, fistulae, perforation), bowel resection (proctosigmoidectomy), and home (routine) versus nonhome discharges. Nonhome discharges included discharges to skilled nursing facilities, acute care hospitals, and home health care. Home vs. nonhome discharges were already recorded within predefined discharge variables in the NIS. In-hospital complications were defined using the 10th revision of the International Statistical Classification of Diseases (ICD-10) procedure codes (Appendices).

All outcomes were stratified by race/ethnicity, as White and non-White (this included Black, Hispanic, Asian or Pacific Islander, Native American, or other races as classified by the NIS description of data elements) for simplicity. As recommended by the Agency for Healthcare Research and Quality (AHRQ), weighted data were used for all statistical analyses [13]. We accounted for clustering (HOSP_NIS), weighting (DISCWT), and stratification (NIS_STRATUM) within the Nationwide Inpatient Sample during this study, ensuring the use of only population-representative data. The normality of the data distribution was confirmed for variables of interest using the Shapiro-Wilk test. We used descriptive statistics to summarize continuous and categorical variables. Mean and standard deviation were used for continuous variables, and totals and percentages were used for categorical variables. We used univariate analyses for between-group comparisons, Pearson’s chi-square test for categorical variables, and Student’s t-test for continuous variables, with a P-value of 0.05 considered statistically significant.

Stepwise multivariate logistic regression was used to estimate the adjusted odds ratio (aOR) and 95% confidence intervals (95% CI) to determine the association between race and measured clinical outcomes in patients with acute diverticulitis. All multivariate logistic regression models used a P-value of 0.05 for statistical significance. Multivariate regression models were adjusted for age, sex, elective vs. nonelective admission, insurance status, patient location, median household income, All-Patient-Refined (APR) severity of illness subclass, APR-Risk of mortality, and statistically significant comorbidities (hypertension, diabetes mellitus, tobacco use, heart failure, hyperlipidemia, obesity, coronary artery disease, COVID-19, chronic kidney disease, and chronic obstructive pulmonary disease) between groups. Multicollinearity was assessed using the variance inflation factor (VIF), with variables having VIF>5 excluded from the final regression model [14]. Missing data elements were treated using multiple imputations [15,16]. Trend analyses were performed using the Jonckheere-Terpstra trend tests with statistical significance set at P<0.05. Hospital costs were estimated in U.S. dollars and adjusted for inflation using the gross domestic product (GDP) price index [17]. All statistical analyses were performed using STATA 17/MP software (Stata Corp, LLC, College Station, TX, USA).

## Results

Baseline characteristics

The mean rate of missing data was 0.5%. 'RACE' exhibited the highest rate of missing data (1.1%). Multiple imputations were applied to treat the absent data. Subsequently, a comprehensive analysis of the study cohort was conducted.

During the study period, 1,266,539 hospitalizations for diverticular disease were recorded. Of these, 953,220 were White patients and 313,319 were not from the White race. Detailed baseline characteristics are shown in Table 1. White patients hospitalized for diverticular disease were typically younger, averaging 63.5 years as compared to 66.8 years for patients who were not of the White race (p<0.001). Additionally, a higher proportion of White patients rather than patients from other races were male (394,633 (41.4%) vs. 122,037 (39.2%); p<0.001) and admitted electively (172,533 (18.1%) vs. 42,962 (13.8%); p<0.001). In contrast, patients from other races were more frequently covered by Medicaid (44,830 (14.4%) vs. 57,193 (6.0%); p<0.001) or were uninsured (17,123 (5.5%) vs. 25,736 (2.7%); p<0.001). In addition, a greater percentage of patients from races other than White belonged to the lower quartile (0-25th percentile) of household income (115,811 (37.2%) vs 208,755 (21.9%); p<0.001).

**Table 1:** Baseline characteristics of individuals with diverticular disease according to race Categorical data is presented as total counts and percentages, n(%) Continuous numerical data is summarized as mean +/- standard deviation (SD) White = White Americans; Non-White includes Blacks, Hispanics, Asians, Pacific Islanders, Native Americans, or other races as classified by the NIS description of data elements CKD, chronic kidney disease; COPD, chronic obstructive pulmonary disease; CAD, coronary artery disease

Variable	White race (N= 953,220), n (%) unless otherwise indicated	Non-White races (N=311,319), n (%), unless otherwise indicated	P-value
Age (years), mean ± SD	63.5 ±6.7	66.8 ± 3.6	<0.001
Female	558,586 (58.6)	189,282 (60.8)	<0.001
Elective vs. nonelective admission	172,532 (18.1)	42,962 (13.8)	<0.001
Inpatient mortality	16,205 (1.7)	4,981 (1.6)	0.006
Insurance Payer			<0.001
Medicare	543,335 (57.0)	151,924 (48.8)	-
Medicaid	57,193 (6.0)	44,830 (14.4)	
Private Insurance	326,954 (34.3)	97,443 (31.3)	
Self-Pay	25,736 (2.7)	17,123 (5.5)	
Household Income			<0.001
0–25thpercentile	208,755 (21.9)	115,811 (37.2)	-
26th–50th percentile	261,182 (27.4)	76,584 (24.6)	
51st–75th percentile	251,650 (26.4)	66,311 (21.3)	
76th–100th percentile	231,632 (24.3)	52,924 (17.0)	
Comorbidities			
Hypertension	441,340 (46.3)	148,499 (47.7)	<0.001
Tobacco use	375,568 (39.4)	101,490 (32.6)	<0.001
Obesity	98,181 (10.3)	36,424 (11.7)	<0.001
Hyperlipidemia	314,562 (33.0)	95,264 (30.6)	<0.000
Diabetes types 1 and 2	193,503 (20.3)	93,084 (29.9)	<0.001
Heart failure	114,386 (12.0)	37,358 (12.0)	0.797
CKD	153,468 (16.1)	41,405 (13.3)	<0.001
COPD	203,035 (21.3)	57,905 (18.6)	<0.001
CAD	2,860 (0.3)	311(0.1)	<0.001
COVID-19	1,906 (0.2)	1,557 (0.5)	<0.001
Patient Location			<0.001
“Central” counties with metro areas of ≥1 million people	192,550 (20.2)	138,225 (44.4)	-
“Fringe” counties with metro areas of ≥1 million people	269,761 (28.3)	65,377 (21.0)	
Counties in metro areas with 250,000–999,999 people	208,755 (21.9)	54,792 (17.6)	
Counties in metro areas with 50,000–249,999 people	100,088 (10.5)	19,613 (6.3)	
Micropolitan counties	101,994 (10.7)	18,990 (6.1)	
Not metropolitan or micropolitan counties	79,117 (8.3)	14,632 (4.7)	

Comorbidities

Patients who were not of the White race had a higher prevalence of hypertension (148,499 (47.7%) vs. 441,340 (46.3%]), obesity (36,424 (11.7%) vs. 98,181 (10.3%)), COVID-19 (1,557 (0.5%) vs 1,906 (0.2%)), and diabetes (93,084 (29.9%) vs. 193,503 (20.3%); p<0.0001 for all). On the other hand, White patients showed a higher prevalence of hyperlipidemia (314,562 (33.0%) vs. 95,264 (30.6%)), tobacco use (375,568 (39.4%) vs. 101,490 (32.6%)), coronary artery disease (2,860 (0.3%) vs. 311 (0.1%)), chronic kidney disease (153,468 (16.1%) vs. 41,405 (13.3%)), and chronic obstructive pulmonary disease (203,035 (21.3%) vs. 57,905 (18.6%); p<0.001 for all).

Hospitalization rates

**Figure 1:**
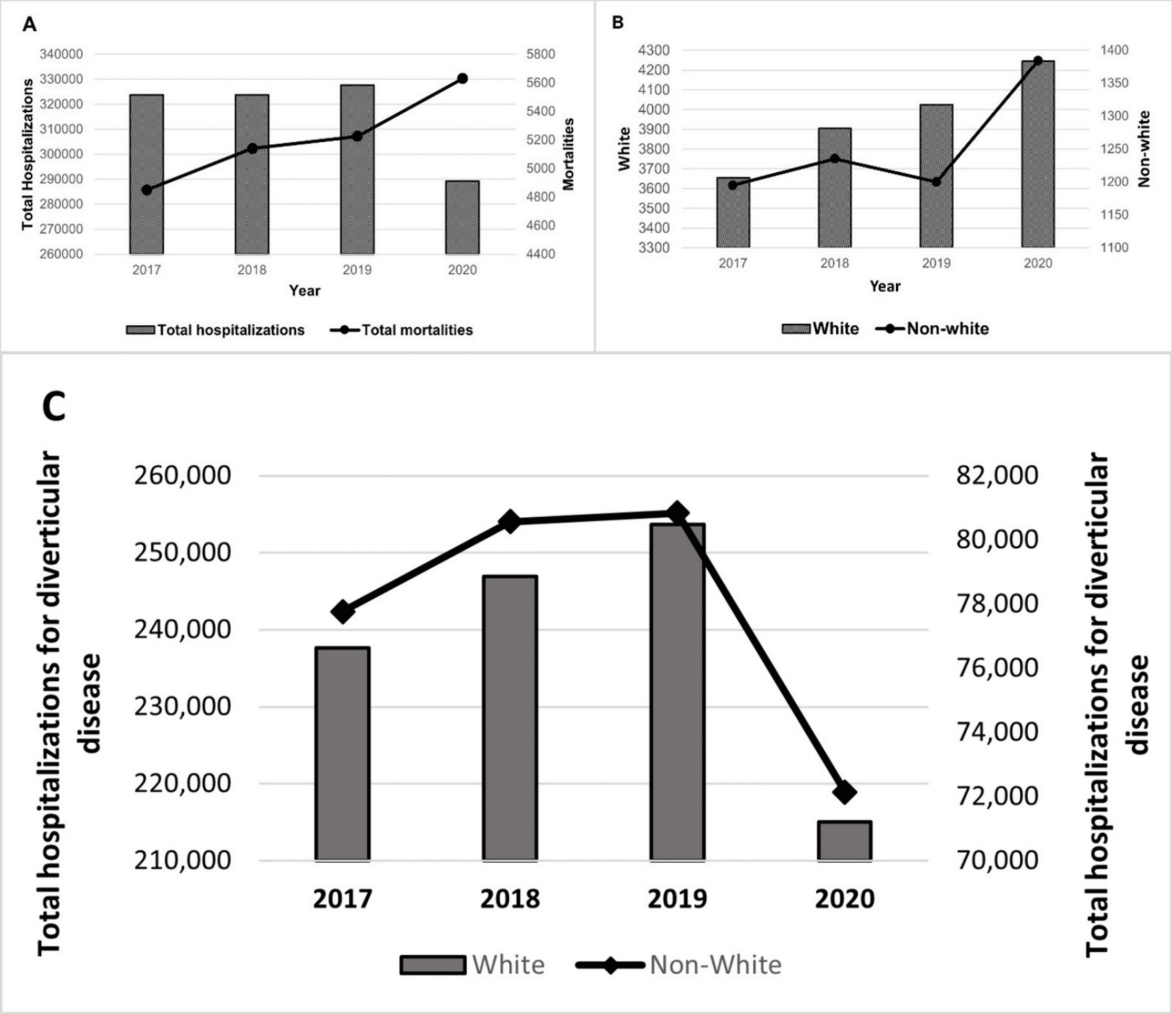
Trends in hospitalization rates and mortality by race A. Trends in hospitalizations and mortality; Ptrend<0.001 B. Trends in mortality among White and non-White patients; Ptrend<0.001 C. Trends in hospitalizations by race; P=0.001 Non-White includes Black, Hispanic, Asian or Pacific Islander, Native American, or other races as classified by the NIS description of data elements.

Between 2017 and 2019, hospitalizations due to diverticular disease increased from 323,764 to 327,770, a 1.2% increase. However, a significant decrease was observed in 2020, with hospitalizations dropping to 289,245, a notable 11.8% decline (Ptrend<0.001; Figure 1A). The mean age of hospitalized individuals decreased from 66.3 years in 2017 to 63.5 years in 2020 (Ptrend=0.007). Examining gender disparities, women’s hospitalizations initially increased from 201,495 in 2017 to 202,715 in 2019 (0.6%), followed by a considerable reduction of 15,645 cases (10.3%) in 2020. In contrast, men’s hospitalizations increased from 147,870 to 151,375 (2.3%) and later declined by 15,645 admissions, reflecting a 10.3% decrease. Diverticular disease hospitalization among White patients increased from 237,600 in 2017 to 253,637 in 2019 (6.7%) and declined by 38,552 (15% decline) in 2020. Hospitalization for DD among patients who were not of the White race increased from 77,065 in 2017 to 80,840 in 2019 (4.9% increase) and decreased to 72,144 (10.8% decline) in 2020. The mean age of those admitted decreased from 66.3 years to 63.5 years over the period (Ptrend=0.007).

Inpatient mortality

**Figure 2:**
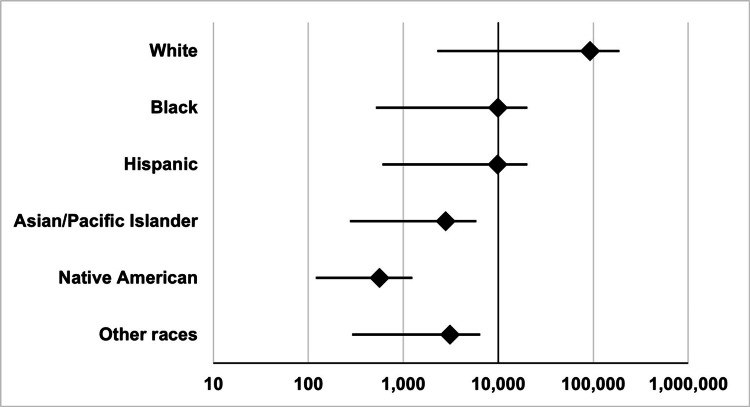
In-hospital mortality according to race Mortality counts are presented in the logarithmic scale

Approximately 21,186 mortalities occurred during the study period (1.6%). White patients had higher mortality than patients of other races (16,205 (1.7%) vs. 5,013 (1.6%); P=0.006; Figure 2). However, no significant difference was found in mortality odds between White and patients who were not from the White race (aOR, 0.953; 95% CI 0.881-1.032; P=0.237). Mortality increased significantly over the study period from 4,850 in 2017 to 5,630 in 2020 (Ptrend<0.001; Figure 1A). Among White patients, mortality increased from 3,655 in 2017 to 4,245 in 2020; among patients from other races, mortality increased from 1,195 in 2017 to 1,384 in 2020 (P<0.001; Figure 1B). Women accounted for 16,205 (76.5%) deaths compared with 4,981 (23.5%) deaths in men. A greater proportion of women died as compared to men during the study (1.6% vs 1.5%; P=0.005).

Resource use burden

**Figure 3:**
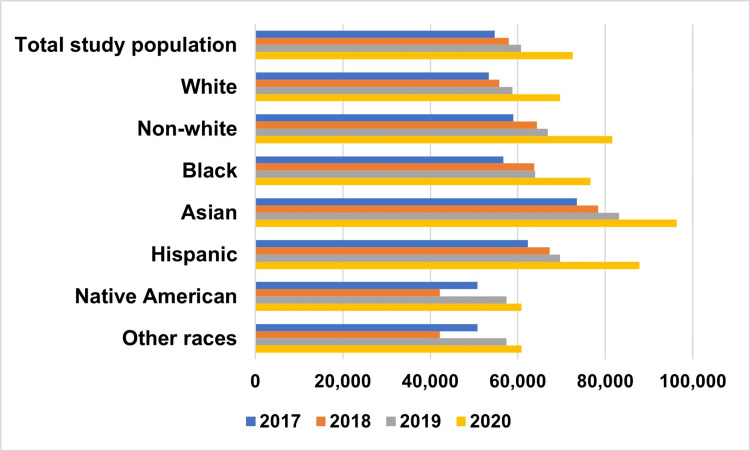
Trends in hospital costs by race Non-White includes Black, Hispanic, Asian or Pacific Islander, Native American, or other races as classified by the NIS description of data elements.

Hospitalizations for diverticular disease accounted for a total of 7,016,718 hospital days during the study period. White patients had 5,234,183 hospital days compared with 1,782,535 hospital days among patients from other races. The mean length of hospital stay was longer for White patients than for the other patients (6.7 ± 3.3 vs. 5.5 ± 1.6 days; P<0.001). A total of 2,102,749 hospital procedures were performed in the study cohort. Diverticular disease incurred US$ 6.8 billion in hospital charges over the study period (Figure 3). After adjusting for inflation, the mean hospital charges increased from US$ 54,705 in 2017 to US$ 72,564 in 2020 (Figure 3; P<0.001). Black, Hispanic, and Asian/Pacific Islander patients had higher mean hospital charges than White patients (67,635 ± 6,700 vs 59,103 ± 3,850; P<0.001). Native American ethnicity was associated with significantly lower mean hospital costs (adjusted mean difference: US$ 9,267; 95% CI: 4,213-14,321; P<0.001).

Complications

The secondary outcomes of the study are shown in Table 2.

**Table 2:** Clinical outcomes in patients with diverticular disease according to race ‡ Adjusted for sex, elective vs. nonelective admission, age, primary payer, all patient refined severity subclass and risk of mortality, location, median household income, hypertension, diabetes mellitus, tobacco use, congestive heart failure, hyperlipidemia, obesity, coronary artery disease, chronic kidney disease, and chronic obstructive pulmonary disease. * Significant at values <0.05 aOR, adjusted odds ratio; CI, confidence interval; THC, total hospital charges; US$, United States Dollar; SD, standard deviation Non-White includes Black, Hispanic, Asian or Pacific Islander, Native American, or other races as classified by the NIS description of data elements.

Outcomes	White (N= 953,220), n (%)	Non-White (N=311,319), n (%)	aOR (95% CI) ‡	P-value*
Primary outcomes				
Inpatient mortality	15,830 (1.7)	5,015 (1.6)	0.95 (0.881 – 1.032)	0.237
Length of stay (days), mean ± SD	6.7 ± 3.3	5.5 ± 1.6	-	<0.001
THC (US$), mean ± SD	59,103 ± 385	67,635 ± 670	-	<0.001
Secondary outcomes				
Bowel abscess	1,370 (0.14)	330 (0.1)	0.52 (0.41-0.71)	0.002
Intestinal fistula	4,950 (0.5)	14,625 (4.7)	0.97 (0.83-1.13)	0.671
Bowel perforation	2,940 (0.3)	875 (0.3)	0.84 (0.60-1.18)	0.315
Need for bowel resection	119,030 (12.5)	28,880 (9.3)	1.28 (0.85-1.38)	0.541
Routine home discharge	654,694 (68.7)	225,555 (72.5)	0.84 (0.79-0.90)	<0.001
Non-home discharge	282,695 (29.7)	80,750 (25.9)	1.21 (1.14-1.29)	<0.001

Compared with patients who are not from the White race, White patients had higher odds of non-home discharge (aOR: 1.21; 95% CI, 1.14-1.29; P<0.001). White patients were less likely to develop bowel abscesses (aOR: 0.52; 95% CI, 0.41-0.71) than patients who were not of the White race. Odds of bowel perforation (aOR: 0.74; 95% CI: 0.49-0.89), routine home discharge (aOR: 0.83; 95% CI: 0.77-0.89; P<0.001), and bowel resection (aOR: 0.90; 95% CI: 0.82-0.99) were all significantly lower in Black patients as compared to White Americans. The odds of bowel resection were significantly higher in Hispanic patients (aOR: 1.13; 95% CI: 1.03-1.25; P=0.012).

## Discussion

The index study highlights racial disparities and trends in diverticular disease hospitalizations across the United States. The findings indicate that disparities transcend various aspects, ranging from hospitalization rates, baseline characteristics of patients, and comorbidities to clinical outcomes following hospitalization. More Whites were hospitalized for diverticular disease than other races combined. Patients who were not from the White race and were hospitalized for diverticular disease over the study period had greater comorbidities such as hypertension, obesity, diabetes, and COVID-19. In contrast, White patients were disproportionately burdened by comorbidities such as hyperlipidemia, tobacco use, and chronic obstructive pulmonary disease. This finding can be juxtaposed with recent research that has reported increased multimorbidity among racial minorities [18,19]. The index study also highlights a surge in diverticular disease hospitalizations from 2017 to 2019, followed by a decline in 2020; potentially, among other factors, a collateral impact of the COVID-19 pandemic. During the initial years, increased awareness and improved diagnostic capabilities may have contributed to the increased number of hospitalizations. However, in 2020, the COVID-19 pandemic led to widespread changes in healthcare use. Many patients avoided hospitals due to fear of contracting COVID-19, resulting in fewer hospital visits and admissions for non-COVID conditions, including diverticular disease. Additionally, healthcare resources were heavily diverted to manage the pandemic, leading to delays in elective procedures and routine care, further reducing hospitalizations for diverticular diseases. Public health measures, such as lockdowns and reduced mobility, may also have impacted the incidence and severity of diverticular disease, contributing to the observed decline in hospital admissions during the pandemic. This rising hospitalization trend could be juxtaposed with findings from prior research, which commonly document an escalating trend in diverticular disease hospitalizations, potentially attributed to aging populations and evolving dietary habits [20-22]. Further research might be instrumental in unraveling the definitive contributors to the observed disparities in hospitalizations for diverticular disease. However, various factors influence racial disparities such as socioeconomic status, structural inequities in the healthcare system, and environmental determinants. Limited access to healthcare services and health education among certain racial groups exacerbates these disparities. Cultural and linguistic barriers can also hinder effective patient-healthcare provider communication, affecting diagnosis and treatment quality. Additionally, variations in genetic predispositions, lifestyle factors, and the prevalence of comorbid conditions across different racial and ethnic groups contribute to the observed disparities in hospitalization rates [23-25].

Hospitalization for diverticular diseases significantly increased during the study period. This increasing incidence is primarily attributed to lifestyle and dietary habits, particularly the consumption of a low-fiber Western diet rich in processed foods and red meat [26,27]. Aging also contributes to the natural weakening of the colonic wall over time [28]. Other significant factors include lack of physical exercise, obesity, and certain medications, such as non-steroidal anti-inflammatory drugs (NSAIDs), which increase the risk. In addition, alterations in gut microbiota due to antibiotic usage or dietary changes can impact the integrity and immunity of the gut, further contributing to the prevalence of these gastrointestinal disorders [29,30]. Few studies have evaluated racial disparities in the incidence or outcomes of diverticular disease. Black Americans diagnosed with diverticulitis tend to have a higher need for immediate or emergency interventions because of recurrent episodes of the disease [31]. They have a higher susceptibility to repeated occurrences of the disease, along with increased rates of complications and fatality [32]. In contrast, the findings of the index study showed that Black Americans had lower odds of bowel perforation or need for bowel resection surgery. They were more likely to be discharged to home health care or skilled nursing facilities compared with routine home discharge. Hispanic descent was associated with a greater risk of bowel resection. No significant difference was observed in the likelihood of bowel fistulae, resection, or perforation between White patients and patients of other races. However, White American descent was correlated with lower odds of abscess formation.

More women than men were diagnosed with diverticular disease during the study period, with the data showing an increasing trend in hospitalization for both men and women. Mortality was greater among women than among men, a finding that correlates with recent data from a Centers for Disease Control (CDC) mortality database spanning January 1999 to December 2016 (adapted by Sell et al. 2020), where 68% of diverticulitis deaths among U.S. citizens over 18 years were women vs. 32% in men. Women were more likely to die from secondary causes of death related to infection, obstruction, and fistulae; conversely, when men died from diverticulitis, the secondary causes were more likely to be procedural or surgical complications [33]. With women accounting for 58.6% of hospitalizations and over 76% of all mortalities, the findings of the index study demonstrate that mortality from diverticulitis among women may have risen since the CDC study and implies a need for more intensive evaluation and treatment of diverticulitis in women.

Diverticular disease has been identified as the fifth most important gastrointestinal disease in the United States from an economic perspective [34]. Over the index study period, significant increments in mean hospitals were recorded for diverticular disease, highlighting its increasing economic burden. Importantly, individuals not belonging to the White race incurred greater mean hospital costs than White individuals. The observed racial disparities in hospital charges for DD hospitalizations can be attributed to several factors. Black, Hispanic, and Asian/Pacific Islander patients often face higher hospital charges due to socioeconomic and systemic issues, such as limited access to early and preventive healthcare, leading to more severe disease presentations and expensive treatments [35-37]. Implicit healthcare provision bias may result in different treatment approaches and lengths of hospital stay [38,39]. Language barriers and cultural differences can further increase resource utilization and extend hospital stays, increasing hospital costs. Conversely, significantly lower hospital costs for Native Americans may be due to the underreporting of health issues, reduced access to healthcare facilities, or differences in healthcare use [40].

Insurance status significantly contributes to these disparities. Patients not of the White race are more likely to be underinsured or uninsured, leading to delayed medical care and more advanced disease stages at hospitalization that require intensive treatment [41]. Insurance type affects the length of hospital stay, treatment availability, and overall costs. Additionally, racial differences in the rates of discharge against medical advice (AMA) contribute to higher costs [42]. Patients who leave AMA often face higher healthcare costs because of complications, higher readmission rates, and noncompliance with follow-up care. Higher AMA discharge rates among racial and ethnic minorities, influenced by mistrust in the healthcare system, cultural misunderstandings, communication barriers, and financial constraints, worsen these cost disparities. Addressing these issues through culturally competent care, improved patient-provider communication, and support systems can help reduce AMA discharge rates and healthcare costs, ensuring equitable outcomes for all racial and ethnic groups.

Strengths and limitations

The index study has considerable strengths, primarily the use of the largest inpatient database in the US, enabling extensive access to inpatient utilization, cost, and outcome data. However, it is not without limitations. The NIS database, being administrative in nature, lacks the granular details of research databases. In addition, the NIS database omits important information, such as patients’ adherence to medication, laboratory results, and the management of patients’ comorbidities, which are factors that are instrumental in influencing the outcomes.

## Conclusions

During the study period, there was a significant increase in hospitalizations, mortality rates, and associated costs for diverticular disease care. This condition remains a major cause of hospital admissions for gastrointestinal disorders, with a concerning upward trend in mortality and resource use. Women accounted for two-thirds of the total mortality from this disease, highlighting the urgent need for improved evaluation and timely treatment in this demographic.

## Appendices

**Table 3:** CCSR and ICD-10 diagnostic codes used in the study CCSR, clinical classification software refined code; ICD-10, International Classification of Diseases, tenth revision

CCSR code	Description
DIG013	Diverticulosis and diverticulitis
ICD-10 code	Description
Perforation of intestine (nontraumatic)	K63.1
Fistula of intestine	K63.2
Bowel abscess	K63.0
Bowel resection	0DBE0ZZ, 0DBE3ZZ, 0DBE4ZZ, 0DBE7ZZ, 0DBE8ZZ, 0DBF0ZZ, 0DBF3ZZ, 0DBF4ZZ, 0DBF7ZZ, 0DBF8ZZ, 0DBG0ZZ, 0DBG3ZZ, 0DBG4ZZ, 0DBG7ZZ, 0DBG8ZZ, 0DBH0ZZ, 0DBH3ZZ, DBH4ZZ, 0DBH7ZZ, 0DBH8ZZ, 0DBK0ZZ, 0DBK3ZZ, 0DBK4ZZ, 0DBK7ZZ, 0DBK8ZZ, 0DBL0ZZ, 0DBL3ZZ, 0DBL4ZZ, 0DBL7ZZ, 0DBL8ZZ, 0DBM0ZZ, 0DBM3ZZ, 0DBM4ZZ, 0DBM7ZZ, 0DBM8ZZ, 0DBN0ZZ, 0DBN3ZZ, 0DBN4ZZ, 0DBN7ZZ, 0DBN8ZZ, 0DTE0ZZ, 0DTE4ZZ, 0DTE7ZZ, 0DTE8ZZ, 0DTF0ZZ, 0DTF4ZZ, 0DTF7ZZ, 0DTF8ZZ, 0DTG0ZZ, 0DTG4ZZ, 0DTG7ZZ 0DTG8ZZ, 0DTH0ZZ, 0DTH4ZZ, 0DTH7ZZ, 0DTH8ZZ, 0DTK0ZZ, 0DTK0ZZ, 0DTK4ZZ, 0DTK7ZZ, 0DTK8ZZ, 0DTL0ZZ, 0DTL4ZZ, 0DTL7ZZ, 0DTL8ZZ, 0DTM0ZZ, 0DTM4ZZ, 0DTM7ZZ, 0DTM8ZZ, 0DTN0ZZ, 0DTN4ZZ, 0DTN7ZZ, 0DTN8ZZ
